# Whole-Genome Sequences of Escherichia coli Isolates from Cocoa Beans Imported from Bolivia

**DOI:** 10.1128/MRA.01516-18

**Published:** 2019-01-17

**Authors:** Hephzibah Nwanosike, Taejung Chung, Lingzi Xiaoli, Megan Condello, Edward G. Dudley, Jasna Kovac

**Affiliations:** a Department of Food Science, The Pennsylvania State University, University Park, Pennsylvania, USA; University of Maryland School of Medicine

## Abstract

Four Escherichia coli isolates were obtained from a batch of cocoa beans imported from Bolivia. The cocoa beans were rejected by a U.S. chocolate manufacturer due to poor microbiological quality. The four isolates were whole-genome sequenced and the sequences analyzed to identify genotypes, serotypes, and virulence and antimicrobial resistance genes.

## Abstract (precis)

Four Escherichia coli isolates were obtained from a batch of cocoa beans imported from Bolivia. The cocoa beans were rejected by a U.S.

## ANNOUNCEMENT

Draft genomes of four Escherichia coli isolates (PS00209, PS00210, PS00211, and PS002120) obtained from a rejected batch of cocoa beans imported from Bolivia to the United States for chocolate production were sequenced and analyzed. The isolates were obtained by plating beans homogenized in a buffer onto E. coli coliform (ECC) agar (CHROMagar). Single colonies were selected and grown in brain heart infusion broth for 15 h at 37°C. DNA was extracted using an E.Z.N.A. bacterial DNA kit (Omega) and quantified using NanoDrop and Qubit instruments. Genomic DNA was used for Nextera XT library preparation (Illumina), according to the manufacturer’s instructions. Libraries were sequenced on an Illumina MiSeq sequencer using a 500-cycle V2 kit with 250-bp paired-end reads.

The isolates PS00209, PS00210, PS00211, and PS00212 were sequenced with 757,824, 667,390, 633,330, and 1,022,068 reads, respectively. Read quality was assessed with FastQC 0.11.7, using default parameters (https://www.bioinformatics.babraham.ac.uk/projects/fastqc/). The reads were trimmed, and poor-quality bases were removed using the default parameters of Trimmomatic 0.38 (1). High-quality reads were assembled *de novo* using SPAdes 3.12.0 with the option “careful” and k-mer sizes of 99 and 127 (2). Draft genomes were checked for quality using default settings in QUAST 5.0.0 (3). The isolates assembled in 5.15-, 4.91-, 5.15-, and 4.76-Mbp-long genomes, with *N*_50_ values of 48,068 bp, 25,175 bp, 34,506 bp, and 48,732 bp and GC contents of 50.44%, 50.75%, 50.47%, and 50.73%, respectively. The average genome coverage was computed using BWA 0.7.17 (4) and SAMtools 1.9 (5) using default settings, resulting in coverages of 30×, 27×, 25×, and 41×, respectively.

Multilocus sequence typing (MLST) genotyping was carried out using EnteroBase 1.1.2 (6, 7). Isolates PS00209 and PS00211 were identified as sequence type 2074 (ST2074), isolate PS00210 as ST118, and isolate PS00212 as ST8369. Virulence genes and serotypes were detected in four draft genomes using VirulenceFinder 2.0 and SeroTypeFinder 2.0, respectively, on the Center for Genomic Epidemiology server, using default settings (8). While no Shiga toxin-encoding genes were detected, isolates PS00209, PS00210, and PS00211 carried virulence genes *eilA* (encoding a virulence gene regulator) (9) and/or *air* (encoding enteroaggregative immunoglobulin repeat protein, an adhesin) (10). These are accessory virulence genes for enteroaggregative E. coli (EAEC) strains that cause noninvasive infection resulting in watery diarrhea. Isolate PS00212 carried the *lpfA* gene (encoding long polar fimbriae, adhesive factors) that had been previously found in EAEC and other pathogenic E. coli strains (11). Nevertheless, the isolates lack a full set of virulence genes that are typically associated with pathogenic E. coli strains. Isolates PS00209, PS00210, PS00211, and PS00212 had O163:H9, H45, O163:H9, and H19 serotypes identified, respectively. Antimicrobial resistance genes were detected in the assembled draft genomes using BTyper 2.3.1, with default settings (12, 13). All four isolates carried genes associated with beta-lactam resistance (*ampH, ampC1, ampC2,* and a penicillin binding protein-encoding gene). Nevertheless, isolates PS00209, PS00210, PS00211, and PS00212 were not phenotypically resistant to ampicillin, as indicated by the 22-, 24-, 22-, and 22-mm zones of inhibition around ampicillin disks (10 μg) in a disk diffusion assay (14).

### Data availability.

The sequence reads and assembled genomes were deposited in NCBI under BioProject number PRJNA487888. The SRA and GenBank accession numbers are SRR7754655, SRR7754656, SRR7754653, and SRR7754654 and QXDN00000000, QXHI00000000, QXHJ00000000, and QXHK00000000 for strains PS00209, PS00210, PS00211, and PS00212, respectively.
